# Modeling the Internal and Contextual Attention for Self-Supervised Skeleton-Based Action Recognition

**DOI:** 10.3390/s25216532

**Published:** 2025-10-23

**Authors:** Wentian Xin, Yue Teng, Jikang Zhang, Yi Liu, Ruyi Liu, Yuzhi Hu, Qiguang Miao

**Affiliations:** 1 School of Information Science and Technology, Dalian Maritime University, Dalian 116026, China; wtxin@dlmu.edu.cn; 2 Institute of Dataspace, Hefei Comprehensive National Science Center, Hefei 231283, China; zhangjikang@163.com (J.Z.); yiliu6@stu.xidian.edu.cn (Y.L.); 3 School of Computer Science and Technology, Xidian University, Xi’an 710071, China; ruyiliu@xidian.edu.cn (R.L.); yuzhihu@stu.xidian.edu.cn (Y.H.); qgmiao@xidian.edu.cn (Q.M.)

**Keywords:** skeleton-based action recognition, multimodal learning, contrastive learning, frequency learning

## Abstract

Multimodal contrastive learning has achieved significant performance advantages in self-supervised skeleton-based action recognition. Previous methods are limited by modality imbalance, which reduces alignment accuracy and makes it difficult to combine important spatial–temporal frequency patterns, leading to confusion between modalities and weaker feature representations. To overcome these problems, we explore intra-modality feature-wise self-similarity and inter-modality instance-wise cross-consistency, and discover two inherent correlations that benefit recognition: (i) Global Perspective expresses how action semantics carry a broad and high-level understanding, which supports the use of globally discriminative feature representations. (ii) Focus Adaptation refers to the role of the frequency spectrum in guiding attention toward key joints by emphasizing compact and salient signal patterns. Building upon these insights, we propose a novel language–skeleton contrastive learning framework comprising two key components: (a) Feature Modulation, which constructs a skeleton–language action conceptual domain to minimize the expected information gain between vision and language modalities. (b) Frequency Feature Learning, which introduces a Frequency-domain Spatial–Temporal block (FreST) that focuses on sparse key human joints in the frequency domain with compact signal energy. Extensive experiments demonstrate the effectiveness of our method achieves remarkable action recognition performance on widely used benchmark datasets, including NTU RGB+D 60 and NTU RGB+D 120. Especially on the challenging PKU-MMD dataset, MICA has achieved at least a 4.6% improvement over classical methods such as CrosSCLR and AimCLR, effectively demonstrating its ability to capture internal and contextual attention information.

## 1. Introduction

Human action recognition (HAR) has emerged as a critical area with wide-ranging applications across sensor-based domains, including consumer-level surveillance [1,2], autonomous driving [3,4], human-computer interaction [5,6], medical rehabilitation [7,8], sports analytics [9,10], and smart city systems [11,12]. Recent HAR methods utilize multimodal sensor data, such as RGB images, depth maps, and optical flow, to capture complementary information and improve recognition accuracy [13]. However, extracting accurate action representations from sensor-derived video is challenging due to background interference and inconsistent lighting, which could distort the relationships between human joints and reduce recognition accuracy [14,15].

Skeleton data provide 3D joint positions, motion details, and topological relationships, offering an efficient and compact representation of spatiotemporal features [16,17]. Although it highlights key attributes of human actions, extracting joint-level information often loses contextual semantics [18]. As a result, it becomes particularly challenging to accurately recognize actions with subtle semantic differences, such as distinguishing between ‘reading’ and ‘writing’, or ‘pointing’ and ‘victory gesture’ [19]. The absence of such semantic information makes it difficult even for experienced researchers to differentiate between similar skeletal patterns, let alone intelligent models [20,21]. As a result, multimodal fusion methods have become increasingly important, especially for fine-grained actions that rely heavily on semantic cues and interaction understanding [22]. Multimodal fusion approaches can generally be divided into two categories: RGB + Skeleton [14,23] and Semantics + Skeleton [24,25]. Although RGB + Skeleton methods achieve high accuracy [26], they require heavy computation and are unsuitable for lightweight applications. In contrast, semantics and skeleton are compact high-level representations, where semantics need some preprocessing, but inference remains more efficient than RGB methods  [27]. Large-scale labeling is costly and error-prone, while self-supervised learning offers a way to reduce such challenges by removing supervision or generating pseudo-supervision [28,29].

Recently, several studies have advanced skeleton-based self-supervised and multimodal learning with novel mechanisms. For example, HiCLR [30] and HYSP [31] explored hierarchical consistency and hyperbolic learning to enhance representation robustness, yet they rely heavily on complex augmentation strategies, which may limit scalability. Skeleton-logoCLR [32] and CStrCRL [33] enhanced feature discrimination through global–local contrastive learning and gated graph modeling, yet they still rely mainly on structural cues without semantic understanding. In contrast, multimodal frameworks such as SAM-Net [34] and CFVL [35] integrated vision–language alignment, achieving better interpretability but incurring heavy computational costs. More recent cross-modal skeleton–language methods, such as ActionGCL [36] and CoCoDiff [37], introduced semantic-guided diffusion and contrastive consistency, but they struggle to maintain stable intra-modal self-similarity, leading to inconsistent representations. These limitations collectively highlight the need for a unified approach that can model both intra-modal self-similarity and inter-modal cross-consistency, motivating the design of our proposed framework.

Current self-supervised contrastive learning methods overlook several key aspects. First, relying on similarity measures in single-modal skeleton data suffers from contextual gaps, missing objects, and entangled spatiotemporal features, which weaken salient feature extraction and cause action misclassification. Second, most methods focus on spatiotemporal pairs while ignoring instance-wise consistency within a modality. Preserving internal structure is crucial for fine-grained details and accurate recognition. Third, point-wise temporal mappings fail to capture global dependencies, and noise, redundancy, and weak joint-movement representation further reduce discriminative power.

To systematically explore the relationship between inter-modal consistency and intra-modal self-similarity, and their combined effect on enhancing action recognition, we propose a novel framework called Modeling Internal and Contextual Attention (MICA). The framework enhances both feature-level and instance-level representations through two core components: the Feature Modulation and the Frequency-domain Spatial–Temporal Block (FreST).

**Figure 1:**
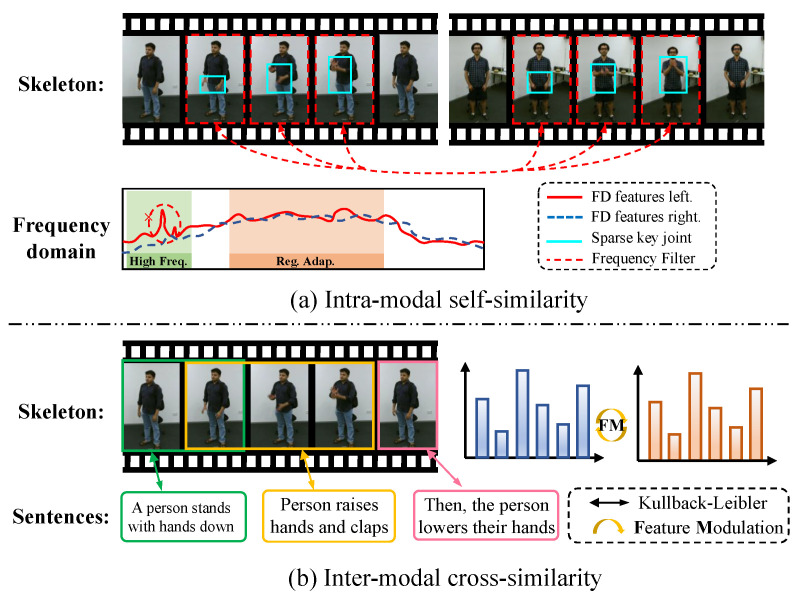
We illustrate two similarity mechanisms for feature association. (**a**) Intra-modal self-similarity: Analyzes skeleton sequences in the frequency domain (high-frequency, adaptive region, etc.) with features like left/right FD features, sparse key joints, and frequency filters to find single-modality associations. (**b**) Inter-modal cross-similarity: Achieves cross-modal association between skeleton sequences and text (e.g., ‘A person stands with hands down’) via KL divergence and Feature Modulation (FM).

Feature Modulation aligns skeletal motion data with corresponding semantic information by minimizing the representational distance between sample features and class-specific anchor points. Such alignment encourages precise decision boundaries and improves discrimination among similar actions. The effectiveness of this mechanism is visually demonstrated in the red sector area of Figure 1. FreST refines motion encoding by transforming skeleton sequences into the frequency domain, highlighting sparse yet informative joint movements. Adaptive filters suppress redundancy while preserving discriminative temporal-spatial features. By combining global and local frequency filtering, FreST captures structural variations and enhances action representations.

Together, Feature Modulation and FreST provide a unified framework for cross-modal alignment and structural modeling, enabling context-aware attention with frequency-domain refinement. Extensive experiments across pre-training, linear evaluation, fine-tuning, and semi-supervised settings demonstrate significant performance gains without requiring extra labeled data. Visual validation further illustrates the skeleton–language action domain. To summarize, our main contributions are threefold:

- We propose a novel self-supervised framework named Modeling Internal and Contextual Attention (MICA), which enhances skeleton-based action recognition by jointly modeling intra-modal self-similarity and inter-modal cross-modal consistency.
- We introduce a Feature Modulation mechanism that constructs a skeleton–language conceptual domain by minimizing the expected information gain between modalities, enabling alignment of action representations in a shared semantic space.
- We design a Frequency-domain Spatial–Temporal Block (FreST) that adaptively filters sparse yet informative joint movements, leveraging global and local frequency filters to capture salient spatial–temporal patterns for fine-grained action recognition.

The remainder of this paper is organized as follows. Section 2 reviews related works on self-supervised learning, contrastive learning, and frequency-based skeleton action recognition. Section 3 introduces the preliminaries, including the skeleton encoder, semantic encoder, and semantic description learning. Section 4 presents the proposed Modeling Internal and Contextual Attention (MICA) framework in detail. Section 5 reports extensive experimental results and ablation studies on multiple benchmark datasets. Finally, Section 6 concludes the paper and discusses possible future research directions.

## 2. Related Works

In this section, we explore key techniques such as self-supervised learning, contrastive learning, multimodal contrastive learning, and frequency feature learning in skeleton-based action recognition.

**Self-supervised Learning with skeleton** aims to learn discriminative feature representations from unlabeled data to reduce the dependence on labeled data. Many efforts have been devoted to designing self-supervised learning frameworks to extract skeleton spatiotemporal motion features for benefiting recognition. Specifically, MS2L [38] performs motion prediction modeling by predicting future sequences, while integrating multi-task and jigsaw puzzle [39,40] recognition to solve the overfitting problem of single-task reconstruction. In addition, recent multimodal self-supervised frameworks such as SeBiReNet [41] and HiCo [42] have explored cross-modal consistency and hierarchical feature learning, providing valuable insights into the design of multi-stream skeleton representation models. However, it is difficult for these methods to compete with advanced supervised methods [43,44,45].

**Skeleton-based Contrastive learning** has demonstrated remarkable performance advantages in self-supervised model pre-training. It effectively enhances the discriminative ability of feature representations and improves model performance in downstream tasks. Contrastive learning has been popularized in skeleton-based action recognition recently, such as SkeletonCLR [28], AimCLR [46], ActCLR [47], and others [48,49]. In addition to contrastive paradigms, non-contrastive self-supervised methods have also shown great potential. For example, [17,50,51,52] learn spatiotemporal representations through masked auto-encoding without using contrastive objectives, effectively capturing fine-grained motion dynamics and frequency-aware structural dependencies in skeleton sequences. However, the information bottleneck (e.g., contextual gap and missing interaction objects) of single-modal skeleton data, and the complete entanglement of information caused by spatiotemporal modeling networks, contribute to the misclassification of similar actions.

**Semantic Augmentation Strategies on Multimodal Skeleton Action Recognition** have brought significant benefits to addressing the aforementioned information gap problem. Approaches such as CLIP [27] and ALIGN [53] achieve cross-modal understanding by learning to compare text and images. Integrating skeletal and linguistic semantics has become a powerful approach to enhance action recognition. Recent methods incorporate descriptive language into skeleton features using prompts, label embeddings, or generated text to enrich contextual understanding. For example, SMIE [54], SA-DVAE [55], and Text-CLS-Transformer [56] align skeleton and text spaces through mutual information maximization, variational modeling, or prompt-based joint embedding, while HSARL [57] introduces motion semantics from language models to improve generalization. Other approaches, such as LPSR [58], ActionGCL [36], and CoCoDiff [37], use contrastive objectives and latent diffusion to enforce consistency between skeleton representations and language embeddings, enhancing discrimination for ambiguous actions. At the structural level, CrossGLG [59], Neuron [60], and LGGT [61] construct skeleton–text association matrices guided by semantic priors to improve spatial–temporal modeling, and methods like GAP [24], MMFR [25], SAM-Net [34], CFVL [35], and SAT-GCN [62] further align inter-joint and inter-class structures using generative prompts and motion cues.

**Frequency Feature Learning** effectively addresses the inherent noise and redundancy present in skeleton data, thereby enhancing the ability to express salient information. DCT [63] introduced a frequency-domain learning approach, showcasing its effectiveness and advantages in a range of tasks (e.g., classification, detection, and segmentation). Frequency-domain compressed representation contains rich patterns for action recognition tasks. Frequency-domain MLPs [64] utilize MLPs in the frequency domain to address point-wise mappings and information bottlenecks in prediction tasks. Nonetheless, the frequency representation learning approach has seen limited application in skeleton-based action recognition.

**Summary and Distinction from Existing Works.** Despite remarkable progress, existing multimodal and skeleton contrastive learning methods still face two key limitations. First, most CLIP-based frameworks (e.g., ActionGCL [36], CoCoDiff [37]) rely on direct similarity maximization between modalities, which often leads to weak intra-modal consistency and insufficient control over semantic alignment. Second, recent frequency-domain methods (e.g., DCT [63], Frequency-MLPs [64]) improve signal compactness but treat frequency patterns as static representations, lacking adaptability to motion-dependent variations. In contrast, our proposed Modeling Internal and Contextual Attention (MICA) introduces two complementary modules: (1) Feature Modulation (FM), which models expected information gain to achieve fine-grained skeleton–language alignment while preserving intra-modal feature similarity; and (2) Frequency-domain Spatial–Temporal Block (FreST), which performs adaptive global–local frequency filtering on sparse joints to retain discriminative and context-aware spectral cues. Together, these designs provide a unified solution that simultaneously strengthens semantic alignment and spectral discrimination, two aspects that have rarely been optimized jointly in previous works.

## 3. Preliminaries

This section introduces foundational knowledge for semantic-guided skeleton-based action recognition, focusing on three key components: the skeleton encoder, the semantic encoder, and semantic description learning. These elements collectively form the basis of frameworks that align body motion and semantic meaning for improved action understanding.

### 3.1. Skeleton Encoder

Given a sequence of human body joints in 2D or 3D coordinates, the skeleton can be structured as a graph G=(V,E), where $V=(v_{1},v_{2},\ldots ,v_{N})$ denotes a set of *N* joints (nodes), each $v_{i}$ representing the 3D coordinate of the *i*-th joint in the human body, and E defines the bones (edges) connecting them. For this undirected graph, an adjacency matrix $A\in R^{N\times N}$ is used, where each entry $A_{i,j}=1$ if joints $v_{i}$ and $v_{j}$ are directly connected, and 0 otherwise. The action sequence is represented by the joint feature set $X=\{x_{t,n}\in R^{C}|1\leq t\leq T,1\leq n\leq N\}$, where $x_{t,n}$ denotes the feature vector of joint $v_{n}$ at frame *t*, and *C* represents the number of input channels (e.g., the 3D coordinates (x,y,z) or other motion-related attributes such as velocity or confidence scores). The overall input can be written as a tensor $X\in R^{T\times N\times C}$. With *X* representing temporal features and *A* capturing the spatial structure, a typical graph convolutional layer performs the update as follows:

(1) $$X^{l+1}=\sigma \left({\tilde{\Lambda }}^{-\frac{1}{2}}\tilde{A}{\tilde{\Lambda }}^{-\frac{1}{2}}X^{l}W^{l}\right),$$

where $\tilde{A}=A+I$ includes self-loops to preserve node features, and $\tilde{\Lambda }$ is the corresponding degree matrix, whose diagonal element ${\tilde{\Lambda }}_{ii}=\sum_{j}{\tilde{A}}_{ij}$ represents the number of connections (including self-loops) of node $v_{i}$. The function σ(·) applies a non-linear activation, and $W^{l}\in R^{C_{l}\times C_{l+1}}$ is the trainable weight matrix at layer *l*. The skeleton encoding process can be simplified using the following formula:

(2) $$S=E_{s}\left(S_{0}\right),$$

where $S_{0}\in R^{T\times N\times C}$ is the raw input tensor of joint features over time, $E_{s}\left(\cdot \right)$ is the learnable skeleton encoder that fuses spatial graph convolutions with temporal modeling to capture joint dependencies, and *S* is the resulting compact spatiotemporal embedding for downstream tasks.

### 3.2. Semantic Encoder

Semantic action recognition transforms a textual description $T_{0}$ (the raw input text) into an embedding vector *Z* by applying a semantic encoder $E_{t}$, where $E_{t}$ refers to a pretrained transformer model such as CLIP or BERT. In this work, we adopt the CLIP text encoder because it provides a well-aligned multimodal embedding space that bridges visual and linguistic semantics. Unlike generic language models, CLIP has been trained on large-scale paired image–text data, enabling it to capture fine-grained action semantics and contextual cues that are crucial for skeleton–language alignment. This characteristic makes it particularly suitable for enhancing cross-modal consistency in self-supervised action recognition. The transformation is expressed as:

(3) $$Z=E_{t}\left(T_{0}\right),\;Z\in R^{d},$$

where *d* denotes the size of the semantic feature space used to match text with visual or skeletal information. Input descriptions can vary in form, including action names, synonymous expressions, structured part-based templates, or rich natural-language paragraphs.

### 3.3. Semantic Description Learning

To integrate semantic guidance into skeleton-based action representation, various strategies have been explored under different supervision paradigms. Despite differences in data and objectives, they aim to align skeleton and text features in a shared space, typically using contrastive learning that pulls positive pairs closer and pushes negatives apart. A typical bidirectional alignment is formulated as:

(4) $$\begin{array}{cc} \; & p_{S\to Z}\left(S_{i}\right)\;=\;\frac{\exp\left(\mathrm{sim}(S_{i},Z_{i})/\tau \right)}{\sum_{j=1}^{B}\exp\left(\mathrm{sim}(S_{i},Z_{j})/\tau \right)},\\ \; & p_{Z\to S}\left(Z_{i}\right)\;=\;\frac{\exp\left(\mathrm{sim}(Z_{i},S_{i})/\tau \right)}{\sum_{j=1}^{B}\exp\left(\mathrm{sim}(Z_{i},S_{j})/\tau \right)}, \end{array}$$

where sim(·) is cosine similarity and τ is the temperature parameter. These probabilities are optimized using the Kullback–Leibler divergence:

(5) $$L_{\mathrm{KL}}\;=\;\frac{1}{2}\;E_{\left(S,Z\right)}[\mathrm{KL}\left(p_{S\to Z}\;∥\;y_{S\to Z}\right)\;+\;\mathrm{KL}\left(p_{Z\to S}\;∥\;y_{Z\to S}\right)],$$

where $y_{S\to Z}$ and $y_{Z\to S}$ are one-hot targets indicating positive pairs. In low-label or label-free settings, textual descriptions generated by large language models serve as weak supervision to guide representation learning. Finally, the overall training objective integrates the semantic contrastive loss with task-specific terms:

(6) $$L_{\mathrm{total}}=L_{\mathrm{cls}}+\alpha L_{\mathrm{KL}}$$

where Lcls is the cross-entropy loss for action classification. The hyperparameter α balances the contribution of the KL divergence. In summary, semantic description learning enriches skeleton-based action recognition by integrating structured or unstructured language as auxiliary supervision.

## 4. Methodology

In this section, we introduce MICA, which explores inter-modal consistency and intra-modal self-similarity and their correlations, benefiting recognition. MICA consists of Feature Modulation (FM) strategy (Section 4.1) combined with a Frequency-domain Spatial–temporal Block (FreST) method (Section 4.2). In addition, SkeletonCLR is introduced to encourage representations of different skeleton sequences to be pushed apart.

### 4.1. Feature Modulation

**Figure 2:**
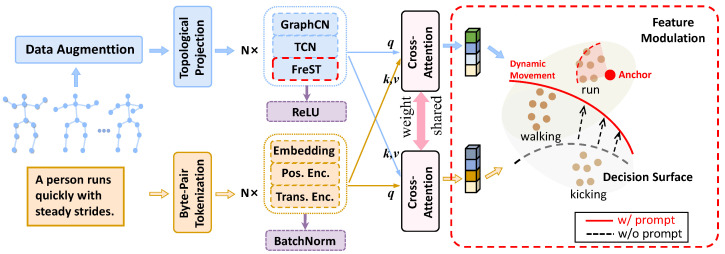
The overall structure of MICA, which is the cross-modal dual-encoder structure, consists of feature modulation and modality harmonizer. Feature modulation introduces text semantic information. The modality harmonizer solves the modal imbalance problem by cross-attention. Additionally, FreST is appended to the end of the skeleton encoder to capture frequency-domain features.

The overall framework of the proposed method includes two branches, as depicted in Figure 2. Within the skeleton branch, the input skeleton sequence undergoes data augmentation and topology mapping, passing through a stack of *N* layers of GCN blocks, which incorporate the proposed frequency-domain spatiotemporal blocks, to encode and generate the skeleton feature representation. Similarly, in the lower branch, the text is processed through Byte-Pair Tokenization and then encoded into embeddings utilizing the CLIP text encoder.

Specifically, we employ the text encoder from the CLIP ViT-B/32 model to obtain sentence-level embeddings. Each action description or generated prompt sentence is first tokenized and then mapped into a 512-dimensional embedding space. These embeddings serve as semantic anchors for cross-modal alignment with skeleton features in the Feature Modulation module. During training, the CLIP encoder remains frozen to preserve its pretrained semantic structure while minimizing the expected information gain between modalities.

To elaborate further, we formulate the process of distillation as an optimization problem, where the text embedding P(X) is the true distribution, which is generated by a pretrained encoder. Q(X) is an approximate distribution used to fit P(X), which is encoded by a learnable graph convolutional neural network. The loss is defined as the expected information gain of P(X) with respect to Q(X), which measures the difference between two distributions.

The process of distillation can be formulated as an optimization problem where we aim to minimize the difference between two distributions. Specifically, the text embedding P(X) represents the true distribution, which is generated by a pretrained encoder. On the other hand, Q(X) is the approximate distribution, which we seek to learn and fit to P(X), using a graph convolutional neural network (GCN).

The goal is to bring Q(X) closer to P(X) by minimizing the discrepancy between these two distributions. This discrepancy is quantified by a loss function, often expressed in terms of the expected information gain (or Kullback–Leibler divergence) between P(X) and Q(X). The KL divergence, denoted as $D_{KL}$(P(X)∥Q(X)), measures how much information is lost when Q(X) is used to approximate P(X). The schematic code is shown as Algorithm 1. The loss function can be formally expressed as:

(7) $$\begin{array}{cc} L_{KL} & =E_{P(X)}\left[logP(X)-logQ(X)\right]\\ \; & =D_{KL}\left(P\left(X\right)∥Q\left(X\right)\right) \end{array}$$

**Algorithm 1** Pseudocode of FM in a PyTorch-like style1:z_q, z_k, z_t: query/key embeddings and text embedding. (B×C)
2:queue_a: queue of N keys (C×N)
3:tau_s, tau_t: temperatures for student/teacher (scalars)4:* * 5:noise_for_q = torch.randn_like(q) × noise_std   # Gauss noise6:noise_for_t = torch.randn_like(z_t) × noise_std7:* * 8:l_a = torch.mm(z_q + noise_for_q, queue_a)   # compute similarities9:l_b = torch.mm(z_q + noise_for_q, z_t + noise_for_t)10:* * 11:loss_kl = loss_kld (l_b/tau_s, z_t/tau_t)12:* * 13:def  14:loss_kld (inputs, targets):15:       inputs, targets = F.log_softmax(inputs, dim = 1), F.softmax(16:       targets, dim = 1)17:       return F.kl_div(inputs, targets, reduction = ’batchmean’)

The Feature Modulation (FM) module enhances action discrimination by adaptively emphasizing discriminative motion cues and suppressing redundant or highly correlated patterns across channels and temporal frequencies. By modulating feature responses conditioned on learned frequency–semantic representations, FM helps to separate subtle inter-class variations (e.g., ‘drinking’ vs. ‘eating’), which often share similar motion trajectories but differ in temporal dynamics or joint coordination. This selective recalibration strengthens the representation’s sensitivity to class-specific motion signatures, thereby improving the distinction between visually or kinematically similar actions.

### 4.2. FreST

Skeleton data usually consists of multiple temporal nodes, reflecting the motion trajectories of different human joints over time. The change frequency of these nodes can reveal key features of certain actions (e.g., speed, rhythm, and periodicity of actions). Specifically, the input skeleton sequence $x\in R^{N\times C\times T\times V}$ passes through the Frequency Spatial Block (FreS) and Frequency Temporal Block (FreT), respectively, with feature optimization via their built-in adaptive frequency-domain filtering. FreS and FreT do not change the dimension of the input sequence. To avoid spatial–temporal feature coupling interference [14,17,21], which occurs when spatial topology and temporal motion cues are entangled within a single representation, a corresponding spatial or temporal decoupling module is added before each frequency-domain module. Such coupling can blur discriminative temporal dynamics with static spatial patterns, thereby reducing filtering precision. By decoupling, the module first separates the skeleton sequence’s spatial topological features (e.g., relative joint positions) and temporal dynamic features (e.g., joint trajectories), then feeds the resulting single-dimensional representations into the subsequent frequency-domain module. This separation allows adaptive filters to focus more effectively on domain-specific variations, ultimately improving the accuracy of frequency-domain feature extraction.

**Figure 3:**
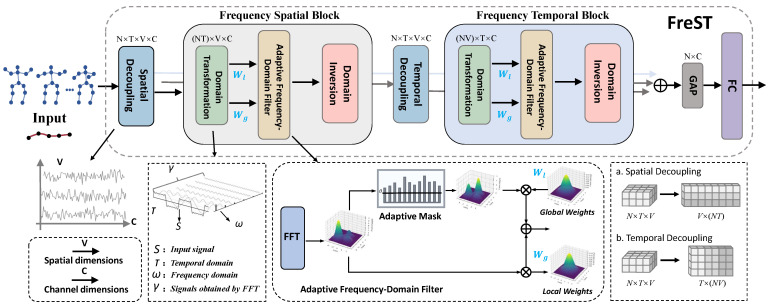
The overall overview of Frequency-domain Spatial-temporal block (FreST): the Frequency Spatial Block captures spatial dependencies by performing adaptive frequency-domain filtering in the spatial dimension; the Frequency Temporal Block captures temporal dependencies by performing adaptive frequency-domain filtering in the spatial dimension.

As shown in Figure 3, the FreS module will be introduced below, and the working mechanism of the FreT module is the same. Specifically, after the input sequence is processed by the spatial decoupling module, its dimension is transformed from $R^{N\times T\times V\times C}\to R^{(NT)\times V\times C}$. In this context, the spatial domain refers to the coordinate space spanned by all skeletal joints, where each node $v_{i}\in V$ corresponds to a specific physical joint location in the human body. The spatial relationships among these joints are defined by the adjacency matrix A, which encodes the topological structure of the human skeleton. Subsequently, we convert the input x[n], where $x\left[S\right]\in R^{(NT)\times V\times C}$ for the spatial branch and $x\left[T\right]\in R^{(NV)\times T\times C}$ for the temporal branch, into the frequency domain F by:

(8) $$\begin{array}{cc} F(\omega ) & =\sum_{n=0}^{N-1}x\left[n\right]\cdot e^{-j\frac{2\pi }{N}\omega n}\\ \; & =\sum_{n=0}^{N-1}x\left[n\right]\left(\cos\left(\frac{2\pi }{N}\omega n\right)-j\sin\left(\frac{2\pi }{N}\omega n\right)\right) \end{array}$$

where F(ω) is the signal (spectrum) in the frequency domain and represents the component of the signal at frequency ω, *t* is a temporal variable, and *j* denotes the imaginary unit. Then, we define the 1D FFT operation in Equation (7) as: $F=F\left[S\right]\in R^{T\times C}$. ${\hat{E}}_{i,j}$ is defined as the normalized energy, calculated as:

(9) $$\begin{array}{c} {\hat{E}}_{i,j}=\frac{{\left|X_{i,j}\right|}^{2}}{media(E)+\epsilon } \end{array}$$

where $E_{i,j}$ denotes the energy of the individual frequency component at (i,j), median(E) is the median value of all frequency component energies, and ϵ is a small constant (e.g., $\epsilon =10^{-8}$) introduced to avoid numerical instability caused by zero denominators. This normalization ensures that the energy values are scaled relative to the central tendency of the energy distribution, facilitating consistent thresholding across different input signals.

(10) $$\begin{array}{c} F_{\mathrm{mask}}=F\left(\omega \right)⊙I\left({\hat{E}}_{i,j}>\tau \right) \end{array}$$

where ⊙ denotes element-wise multiplication, and I(·) is the indicator function that generates a binary mask matrix: $I({\hat{E}}_{i,j}>\tau )=1$ if the normalized energy ${\hat{E}}_{i,j}$ exceeds the threshold τ, and 0 otherwise. Through this operation, frequency components with normalized energy above τ are retained in $F_{\mathrm{mask}}$, while those below the threshold are filtered out. Notably, the threshold τ is dynamically adjusted based on the temporal characteristics of the specific action being processed (e.g., motion intensity, frequency bandwidth of key action features), ensuring that critical frequency information (e.g., discriminative motion patterns) is preserved while suppressing high-frequency noise and redundant components that are irrelevant to the action semantics.

After applying adaptive filtering to the frequency-domain data, we introduce two types of learnable filters to further model frequency-domain characteristics. The global filter $W_{g}$ operates directly on the original frequency-domain data F(ω), enabling the model to capture global frequency correlations that may span the entire spectral range. In contrast, the local filter $W_{l}$ is applied to the adaptively filtered result $F_{\mathrm{mask}}$, focusing on learning discriminative patterns within the frequency components deemed important by the adaptive thresholding step. Both filters are parameterized to handle complex-valued frequency-domain data, with their mathematical formulations given by:

(11) $$\begin{array}{c} W_{g}=W_{g}^{r}+jW_{g}^{i},\;W_{l}=W_{l}^{r}+jW_{l}^{i} \end{array}$$

where $W^{r}$ and $W^{i}$ denote the real and imaginary parts of the complex-valued filters, respectively, and *j* is the imaginary unit satisfying $j^{2}=-1$. To initialize these filters in a stable manner, both $W_{g}$ and $W_{l}$ are sampled from a zero-mean Gaussian distribution with variance $\sigma^{2}$ (e.g., $\sigma^{2}=0.01$), ensuring that initial filter responses are moderate and avoid saturating subsequent computations.

The application of these filters to the frequency-domain data is defined as:

(12) $$\begin{array}{c} F_{G}=W_{g}⊙F\left(\omega \right)\\ \;F_{L}=W_{l}⊙F_{\mathrm{mask}} \end{array}$$

where $F_{G}$ represents the globally filtered frequency features, capturing broad spectral patterns across the entire frequency domain, while $F_{L}$ denotes the locally filtered features, which focus on the adaptively selected critical frequency components.

Finally, to integrate both global context and local discriminative details, the output frequency features are computed as the sum of the two filtered results: $F_{\mathrm{output}}=F_{G}+F_{L}$. This integration strategy ensures that the model preserves both coarse-grained global frequency characteristics and fine-grained local details, enhancing the representation capacity for complex spatiotemporal patterns in action recognition tasks.

**Modality Harmonizer.** After obtaining the enhanced frame-level feature $F_{v}^{\mathrm{enh}}$ and the enhanced word-level feature $F_{q}^{\mathrm{enh}}$, we apply cross-attention layers between $F_{v}^{\mathrm{enh}}$ (as the query) and $F_{q}^{\mathrm{enh}}$ (as the key and value) to facilitate interaction and alignment between modalities. The final aligned feature $F\in R^{L_{v}\times D}$ is then derived using the standard cross-attention mechanism.

**Design Rationale of FreST.** The Frequency-domain Spatial–Temporal (FreST) block leverages the intrinsic spectral sparsity of skeletal motion to selectively retain action-relevant components while suppressing unstable oscillations. Concretely, an adaptive mask is formed using a threshold τ computed from the median spectral energy of each input sequence, so that filtering automatically tightens for noise-dominated spectra and relaxes for clean, low-frequency motions. FreST uses two learnable complex-valued filters: a global filter $W_{g}$ that captures sequence-level rhythmic regularities and a local filter $W_{l}$ that focuses on joint-wise short-range variations. This dual-filter parameterization aligns coarse temporal rhythm with fine spatial–temporal details in a single representation. Compared with time-domain smoothing, which operates on strongly correlated samples and often blurs subtle class-discriminative dynamics, frequency-domain selection compacts signal energy, stabilizes optimization, and preserves fine-grained motion signatures, thereby strengthening separability for visually or kinematically similar actions.

**Domain Inversion Module.** The Domain Inversion module functions as a core bridge between the frequency and spatial/temporal domains. It first converts the skeleton features back from the frequency domain to the original spatial or temporal space, ensuring that frequency-enhanced information can be seamlessly integrated into subsequent processing. Meanwhile, it refines the filtered frequency components by suppressing noise and preserving valid motion frequencies, thus improving the quality and stability of the reconstructed skeleton features.

### 4.3. Skeleton Instance Contrastive Loss

We employ identical skeleton encoders to enable contrastive learning at the feature-wise level between upward and downward skeleton modalities. Specifically, given an original skeleton sequence *S*, we apply two different augmentations, T and T′, to generate the query and key samples, denoted as *x* and $\hat{x}\in R^{C\times T\times V}$, where *C*, *T*, and *V* represent the number of channels, frames, and nodes, respectively. A query encoder $f_{\theta_{q}}$ and a momentum-based key encoder $f_{\theta_{k}}$ are employed. Following this, global average pooling (GAP) is applied to derive the query embeddings *z* and key embeddings $\hat{z}$. To optimize the encoder representations and enforce similarity between positive pairs while distinguishing negatives, we adopt the InfoNCE loss as our training objective:

(13) $$\begin{array}{c} \;L_{info}=-log\frac{exp(\hat{z}\prime \cdot z\prime /\tau )}{exp\left(\hat{z}\prime \cdot z\prime /\tau \right)+Z\left(\hat{z}\prime \right)+exp\left(\tilde{z}\prime \cdot z\prime /\tau \right)} \end{array}$$

where · represents the dot product of calculating the similarity between the two normalized embeddings, and τ is the temperature hyperparameter (set to 0.2 by default). $Z\left(v\right)=\sum_{i=1}^{K}exp\left(v\cdot m_{i}/\tau \right)$ represents the similarity between embedding in view *v* and memory queue *Q*, and *K* represents the total number of samples stored in the queue *Q*. The parameters of the query encoder $f_{\theta_{q}}$ are updated by gradient backpropagation, while the parameters of the key encoder $f_{\theta_{k}}$ are updated to the moving average of the query encoder, which can be expressed as:

(14) $$\begin{array}{c} \theta_{k}=m\theta_{k}+\left(1-m\right)\theta_{q} \end{array}$$

where m∈[0,1) is a momentum coefficient, usually close to 1, to maintain consistency in embedding in memory queues. Finally, the loss used to optimize the encoder can be formulated as:

(15) $$\begin{array}{c} L=L_{info}+\lambda L_{KL} \end{array}$$

where λ is a hyperparameter to balance the different sample pairs. Additionally, we incorporate a Frequency-domain Signal Enhancement module (FreST) following the skeleton encoder. FreST enhances the model’s ability to retain critical action information in the latent space by extracting sparse human joint information, which captures compressed signal energy. This feature extraction effectively boosts action recognition performance by emphasizing the most informative skeletal features.

## 5. Experiments

### 5.1. Datasets

**NTU RGB + D 60 (NTU 60):** [16] comprises 56,880 action samples from 40 subjects (ages 10 to 35) captured by Kinect v2. It provides four synchronized modalities: high-resolution RGB videos (1920 × 1080), depth maps, infrared frames (512 × 424), and 25-joint 3D skeleton data, covering 60 action classes including daily activities, health-related behaviors, and interpersonal interactions. Each action is recorded from three horizontal angles (−45°, 0°, +45°) and two subject-facing directions, resulting in six viewpoints. Additionally, 17 different camera setups introduce spatial diversity. Following the official evaluation protocols, two standard benchmarks are defined. In the cross-subject setting, samples from 20 subjects (a total of 40,320 sequences) are used for training, while the remaining 20 subjects (16,560 sequences) are held out for testing. In the cross-view setting, data captured by cameras 2 and 3 (37,920 samples) constitute the training set, and those recorded by camera 1 (18,960 samples) are used for testing.

**NTU RGB + D 120 (NTU 120):** [65] extends NTU 60 to 114,480 samples over 120 action classes, recorded via Kinect v2 from 106 subjects of varied ages and cultures. Samples span 96 environments and 155 camera views, offering RGB, depth, infrared, and 25-joint skeleton modalities. Two evaluation protocols are defined: Cross-Subject and Cross-Setup. Specifically, under the cross-subject protocol, samples from 53 subjects (approximately half of the participants) are used for training, while those from the remaining 53 subjects are reserved for testing. Under the Cross-Setup protocol, data captured in even-numbered setups constitute the training set, whereas samples recorded in odd-numbered setups are used for testing.

**PKU-MMD:** [66] is a medium-scale dataset designed for continuous action detection and multi-modality human activity analysis, captured with Kinect v2. It includes over 1000 untrimmed video sequences with synchronized RGB, depth, infrared, and 25-joint 3D skeleton data. The dataset is split into two parts with varying detection difficulty: Part I features clearly separated actions (1076 videos, 51 classes, 66 subjects), while Part II includes more challenging overlapping actions (1009 videos, 41 classes, 13 subjects). Under the protocol, Part I uses data from 57 subjects for training and 9 subjects for testing (944 and 132 videos, respectively). Part II serves as an independent test set consisting of 13 unseen subjects, used exclusively to evaluate cross-subject generalization—models are trained on Part I and tested on Part II.

### 5.2. Implementation Details

ST-GCN is adopted as the skeleton encoder. The number of input channels is set to the original 1/4, and the feature dimension is set to 512. For frequent learning, both global filters $W_{g}$ and local filters $W_{l}$ are normal distributions with a standard deviation of 0.02. For data augmentation, spatial *Shear* and temporal *Crop* are utilized to generate different skeleton views. For contrastive setting, we set *K* = 32,768, τ = 0.2, m = 0.999, and λ = 0.01. For optimization, we employ SGD with momentum (0.9) and weight decay (0.0001), training the model for 300 epochs with a learning rate of 0.1. Then, we evaluate our approach by comparing it to other methods across several protocols, including linear evaluation, fine-tuning, and semi-supervised evaluation. All experiments are conducted on PyTorch 1.4.0 using an RTX 3090ti (NVIDIA, Santa Clara, CA, USA). When training on the NTU60 dataset with a batch size of 128, the memory usage of a single RTX 3090 graphics card is 21 GB.

**Linear** Evaluation Protocol. We add a fully connected layer with a Softmax activation function on top of the frozen pretrained model, and train the classifier using supervised learning. The classifier is trained for 120 epochs with an initial learning rate of 5, which is reduced by a factor of 0.1 at the 80th epoch.

**Fine-tuning** Protocol. The fine-tuning protocol adds a linear classifier after the pretrained model and trains the entire model for action recognition tasks. Unlike the linear evaluation approach, the pretrained model remains trainable in this protocol. We use supervised learning to train the entire model and compare its performance with other supervised methods.

**Semi-supervised Evaluation Protocol.** We first pre-train the encoder using all available unlabeled data. Then, we finetune the entire model with a small subset of labeled data, selecting either 1% or 10% of the labeled samples at random. Specifically, we employ this strategy to finetune the model using either 1% or 10% of the labeled data.

**Performance Evaluation Measures.** To ensure a fair and consistent comparison with prior work, we adopt widely used quantitative evaluation measures for skeleton-based action recognition. All reported results are based on Top-1 classification accuracy (%) under the standard cross-subject (xsub) and cross-view (xview) protocols of the NTU RGB + D 60 and 120 datasets. In addition, we assess the quality of the learned representations through multiple evaluation strategies, including linear evaluation, where the pretrained backbone is frozen and a linear classifier is trained on labeled data; k-nearest neighbor (k-NN) evaluation, which measures representation separability in feature space; and fine-tuning, where all model parameters are updated for the downstream task. For semi-supervised experiments, the model is trained on a small labeled subset combined with unlabeled samples to evaluate generalization under limited supervision. All metrics are computed per class and averaged (macro-average) over the dataset. These measures are consistent with recent self-supervised and multimodal skeleton learning benchmarks, ensuring the comparability and reproducibility of our experimental results.

**Fair Comparison Protocol.** To ensure a fair and transparent comparison with existing self-supervised and multimodal contrastive methods, we strictly adopted consistent backbone architectures, augmentation strategies, and training configurations across all experiments. Specifically, all methods use the ST-GCN backbone with identical input modalities and data preprocessing. During pre-training, the backbone parameters are unfrozen and updated jointly with the projection head, while during linear evaluation, the backbone is frozen and only the classifier layer is trained. Data augmentation (Spatial Shear and Temporal Crop), learning rate schedules, and optimizer settings (SGD with momentum 0.9 and weight decay 0.0001) are kept identical across all compared methods.

**Dataset Preprocessing.** Following the preprocessing strategy of SkeleMixCLR, we apply a unified and reproducible pipeline to ensure consistency across datasets. For each frame, 3D joint coordinates are centered at the hip joint and scaled by the average bone length to achieve translation and scale invariance. All skeleton sequences are temporally resampled to 64 frames using linear interpolation. Missing or noisy joints are linearly interpolated from adjacent frames along the temporal dimension, and samples with severely incomplete skeletons (typically more than 30% missing joints) are excluded to ensure data integrity. After preprocessing, the joint coordinates are normalized to the range of [−1,1] for stable model convergence. We also adopt skeleton-specific augmentations such as temporal cropping, joint jittering, and random rotation, consistent with SkeleMixCLR, to enhance generalization and robustness against sensor noise.

**Text Description Generation.** We follow the GAP [24] framework and employ a large-scale language model (GPT-3.5) as a knowledge engine to produce natural-language descriptions of actions. Given each action label, the model automatically generates both global descriptions and body-part-specific descriptions using structured prompts. For example, for the action ‘put on a shoe,’ the model outputs a global narrative (‘The person bends down and puts their foot into the shoe’) and detailed part-level semantics (‘head tilts slightly forward; hand reaches down and grasps the shoe; leg bends at the knee, bringing the foot closer to the hand’). These descriptions are encoded using a pretrained CLIP text encoder, whose embeddings serve as semantic supervision for the skeleton encoder through a multi-part contrastive learning objective, allowing the model to align motion patterns with language-based semantics.

### 5.3. Comparison with State-of-the-Art Methods

We first compare MICA with the advanced State-of-the-art methods. Table 1 shows comparison results of the three stream (e.g., joint, bone, and motion) on NTU RGB + D 60, NTU RGB + D 120, and PKU-MMD dataset using linear evaluation protocol. It is evident that our MICA method achieves state-of-the-art performance across each benchmark, indicating that our approach enables the model to effectively capture internal and contextual attention to learn discriminative feature representations. This achievement underscores the effectiveness of our approach in equipping the model to capture both internal and contextual attention, a capability crucial for learning highly discriminative feature representations. By focusing on modeling internal relationships within actions as well as contextual dependencies between them, our approach enhances the model’s ability to accurately recognize complex action patterns. Importantly, this improvement is achieved without relying on additional labeled data, making our method efficient and adaptable for scenarios where labeled data are scarce. Furthermore, when compared to fully supervised methods like ST-GCN, our MICA method exhibits substantial gains, underscoring its potential to outperform traditional techniques in both accuracy and generalization across different datasets and action recognition tasks.

### 5.4. Ablation Studies

In this section, we conduct ablation experiments on NTU RGB + D 60, NTU RGB + D 120, and PKU-MMD joint modality to better verify the role of different submodules in our framework.

**Effectiveness of FM.** We analyze the impact of $L_{FM}$. Specifically, we apply a forward ablation strategy to assess the FM module. Feature Modulation leverages textual semantics to guide the calculation of distribution differences. As shown in Table 2, experiments using only FM demonstrate excellent performance on popular human action benchmarks. Specifically, the joint modality on the NTU X-sub and X-view datasets achieves improvements of 1.8% and 1% over the baseline, respectively. These results validate the necessity of cross-modal, instance-wise similarity calculations, as well as the feasibility and efficiency of FM. The Frequency-domain Module (FM) effectively filters noise information through adaptive frequency-domain filtering technology; more critically, it can accurately distinguish actions with similar motion patterns from a high-level semantic perspective by means of prompts, as specifically illustrated in the comparative example of ‘run’ and ‘walk’ actions in Figure 2.

**Effectiveness of FreT.** As shown in Table 2, combining FreqT with $L_{FM}$ resulted in a performance improvement of 2.3% under the NTU RGB + D 60 X-sub protocol, increasing from 82.5% to 84.8%. The FreqT strategy effectively enhanced the collaboration between the time domain and frequency domain within the encoder, enabling the model to better capture discriminative features related to actions. On other datasets and protocols, FreqT also demonstrated robust performance gains, indicating its broad applicability across different scenarios and a significant improvement in feature representation capability.

**Effectiveness of FreS.** As shown in Table 2, incorporating FreqS with the baseline $L_{FM}$ consistently boosts performance, with a notable increase from 80.7% to 82.7% on the NTU RGB + D 60 X-sub protocol. This improvement highlights the effectiveness of FreqS in capturing spatial-frequency information crucial for recognizing complex actions. Across other datasets and protocols, we observe that FreqS contributes stable performance gains, demonstrating its generalizability and robustness.

**Effectiveness of $W_{g}$ and $W_{l}$.** As shown in Table 3, we demonstrate the effectiveness of the global filter $W_{g}$ and the local filter $W_{l}$. Removing either $W_{g}$ or $W_{l}$ leads to a performance decrease of 0.7% and 0.5%, respectively, indicating the contribution of each filter to the overall model performance.

**Efficiency Analysis of FreST.** As summarized in Table 3, targeted ablation experiments validate the design of FreST. Removing the adaptive thresholding reduces recognition accuracy, while eliminating the global $W_{g}$ or local $W_{l}$ filter causes further performance drops of 0.7% and 0.5%, respectively, confirming their complementary roles. When the complete FreST module is applied, the overall accuracy improves by 3.5% compared with the baseline, with only 0.18 M additional parameters and 1.72 G FLOPs. These results indicate that the adaptive frequency selection and dual-filter structure achieve a favorable balance between model complexity and recognition performance, providing scalability for large-scale and real-time skeleton-based action recognition.

#### 5.4.1. Performance Comparison

**Linear Evaluation.** As illustrated in Table 4, we compare MICA with state-of-the-art self-supervised methods on NTU-60 and NTU-120 under the linear evaluation protocol. MICA demonstrates superior performance over other methods in both single-stream and multi-stream settings. Specifically, MICA surpasses AimCLR by 7% and 7.5% on the X-sub and X-view protocols, respectively.

**Finetune Evaluation.** Table 4 provides comparisons on NTU RGB + D (60 & 120) under the finetune evaluation protocol, and our method leads 7% and 7.5% in X-sub and X-view protocols, respectively, compared to the more than 3s-AimCLR. The results suggest that our method captures more discriminative features and offers better robustness, further reinforcing its potential for real-world applications in action recognition.

**Semi-supervised Evaluation.** Table 5 shows the comparisons on NTU RGB + D (60 & 120) under Semi-supervised evaluation protocol. In this setting, a portion of the labels is available for training, while the remaining labels are withheld, testing the capacity of the model to generalize from limited labeled data. Specifically, our method leads to 7% and 7.5% in X-sub and X-view protocols, respectively, compared to the 3s-AimCLR.

**Quantitative Results.** To conduct multiple analyses of model complexity, we explore the #Params and FLOPs of our method as shown in Table 3. The addition of $L_{M}$ does not change the number of parameters, but it results in a 1.8% improvement over the baseline, which is significant. The introduction of FreST increases the parameter count by 0.18 M, and considering the performance boost, this additional parameter burden is acceptable.

**Figure 4:**
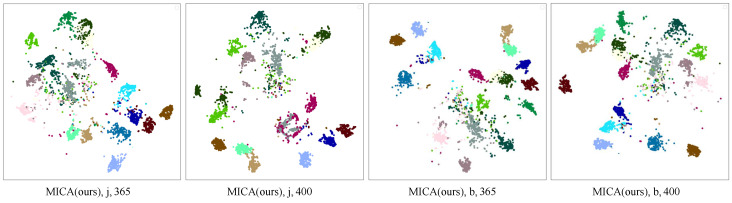
The t-SNE [73] visualization for ambiguous action groups on NTU RGB + D 60 dataset. Different colors indicate different classes.

**Visualization.** Figure 4 shows the t-SNE visualization of MICA and SkeletonCLR on the NTU RGB + D 60 X-sub joint stream, based on 20 randomly selected action classes (different colors indicate different classes). At the abstract level, MICA demonstrates clearer class separation, indicating improved inter-class separability. At the concrete level, intra-class compactness is enhanced, suggesting better clustering within the same class. MICA outperforms SkeletonCLR in feature discrimination, effectively capturing the semantics of the selected action categories.

**Robustness and Sensitivity Analysis.** To further verify the robustness of MICA, we maintained the same random seed and key hyperparameter settings as SkeletonMixCLR (init_seed(2), *K* = 32,768, τ = 0.2, *m* = 0.999) and evaluated multiple CLIP-based semantic encoders under identical training conditions on an RTX 3090 GPU (NVIDIA, Santa Clara, CA, USA). The results summarized in Table 6 show that the framework achieves stable accuracy across different encoders, with ViT-B/32 performing best (84.40%) while maintaining a constant memory usage of 23.12 GB. These findings confirm that the proposed method is robust to encoder variation and insensitive to minor hyperparameter perturbations, ensuring both performance stability and practical efficiency.

#### 5.4.2. Limitations

While our framework achieves competitive results, several limitations remain. First, the performance may degrade when the input skeletons are severely corrupted or noisy, as the feature extraction relies on relatively stable joint trajectories. Incorporating denoising or uncertainty-aware modules could alleviate this issue in future work. Second, although Table 3 reports parameter counts and FLOPs, we note that both the Frequency Modulation (FM) and FreST modules introduce moderate computational and memory overhead, especially when processing long temporal sequences. Nonetheless, these components were designed with lightweight attention operations, maintaining a good balance between accuracy and efficiency. In addition, since all evaluations follow the official cross-subject and cross-view protocols of NTU RGB + D and PKU-MMD, k-fold cross-validation is not applicable in this context, but we acknowledge this as an inherent limitation of the standardized benchmark setting. Finally, our current implementation has been validated on medium-scale benchmarks; scaling to larger datasets or real-time deployment would require further optimization and possibly model compression techniques, which we plan to explore in future work.

## 6. Conclusions

In this paper, we propose an innovative self-supervised framework for skeleton-based action recognition, addressing key challenges in contrastive learning for cross-modal alignment and spatiotemporal feature extraction. Our approach, Modeling Internal and Contextual Attention (MICA), leverages a cross-modal dual-encoder structure with two key components: Feature Modulation and the Frequency-domain Spatial-Temporal block (FreST). Feature Modulation builds a robust skeleton–language feature space by enhancing intra-modality self-similarity and inter-modality instance-wise cross-consistency, thereby addressing the modality imbalance and enriching mutual information exchange. Meanwhile, FreST focuses on the frequency components of sparse key joints, enabling the model to prioritize action-relevant features through compact signal energy. Extensive experiments on the NTU-60, NTU-120, and PKU-MMD benchmarks validated the effectiveness of our approach, demonstrating significant improvements in performance across multiple evaluation protocols, including fine-tuning, linear evaluation, and semi-supervised learning.

## Figures and Tables

**Table 1:** Comparison to the state-of-the-art methods for action recognition accuracy on the NTU RGB + D 60, NTU RGB + D 120, and PKU-MMD I datasets under the linear evaluation protocol. G denotes GCN or ST-GCN, R denotes GRU, T denotes Transformer, and M denotes MAE. J, B, and M denote Joint, Bone, and Motion, respectively.

Method	Publication	Architecture	Modality	NTU60		NTU120		PKU-MMD
		G ‖ R ‖ T ‖ M		X-Sub	X-View	X-Sub	X-Set	Part I
*Linear Evaluation Single-stream Results* (Arranged by Backbone Model and Publish Year)								
SkeletonCLR [28]	CVPR’21	• ‖∘‖∘‖ ∘	J	68.3	76.4	56.8	55.9	80.9
CrosSCLR [28]	CVPR’21	• ‖∘‖∘‖ ∘	J	78.7	84.9	68.7	69.6	-
AimCLR [46]	AAAI’22	• ‖∘‖∘‖ ∘	J	74.3	79.7	63.4	63.4	-
CMD [67]	ECCV’22	∘ ‖•‖∘‖ ∘	J	79.8	86.9	70.3	71.5	-
RVCTL [68]	CVPR’23	∘ ‖∘‖•‖ ∘	J	74.7	79.1	68.0	68.9	-
HYSP [31]	ICLR’23	• ‖∘‖∘‖ ∘	J	78.2	82.6	61.8	64.6	83.8
HiCLR [30]	AAAI’23	∘ ‖∘‖•‖ ∘	J	78.8	83.1	67.3	69.9	73.8
ActCLR [47]	CVPR’23	• ‖∘‖∘‖ ∘	J	80.9	86.7	69.0	70.5	-
SkeAttnCLR [69]	IJCAI’23	• ‖∘‖∘‖ ∘	J	80.3	86.1	66.3	74.5	87.3
DMMG [70]	TIP’23	• ‖∘‖∘‖ ∘	J	82.1	87.1	69.6	70.1	90.7
Skeleton-logoCLR [32]	TCSVT’24	• ‖∘‖∘‖ ∘	J	82.4	87.2	72.8	73.5	90.8
CStrCRL [33]	TCSVT’24	• ‖∘‖∘‖ ∘	J	78.9	84.0	68.8	69.3	-
STHMAE [17]	Sensors’25	∘ ‖∘‖∘‖ •	J	84.3	87.0	74.3	75.6	-
**MICA (Ours)**	This work	• ‖∘‖∘‖ ∘	J	84.4	87.8	71.7	75.4	91.8
3s-CrosSCLR [28]	CVPR’21	• ‖∘‖∘‖ ∘	J + M + B	77.8	83.4	67.9	66.7	84.9
3s-AimCLR [46]	AAAI’22	• ‖∘‖∘‖ ∘	J + M + B	78.9	83.3	68.7	69.6	87.8
3s-CMD [67]	ECCV’22	∘ ‖•‖∘‖ ∘	J + M + B	84.1	90.0	74.0	75.2	-
3s-CPM [71]	ECCV’22	• ‖∘‖∘‖ ∘	J + M + B	84.8	90.9	74.6	76.1	-
3s-ActCLR [47]	CVPR’23	• ‖∘‖∘‖ ∘	J + M + B	85.1	91.4	75.4	76.0	-
3s-FDMAE [52]	SPL’25	∘ ‖∘‖∘‖ •	J + M + B	86.4	90.4	78.9	79.9	92.3
**3s-MICA (Ours)**	This work	• ‖∘‖∘‖ ∘	J + M + B	85.3	90.6	77.4	76.0	93.0

**Table 2:** Exploration of different pre-training settings on the NTU-60, NTU-120, and PKU dataset. FM, FreqT, and FreqS denote Language-guided Feature Modulation, Frequency-domain Temporal block, and Frequency-domain Spatial block, respectively. w/ and w/o mean with and without the corresponding module.

Pre-Training Settings	NTU RGB + D 60		NTU RGB + D 120		PKU-MMD	
	X-Sub	X-View	X-Sub	X-Set	Part I	Part II
w/o pre-training	80.7	85.5	69.0	68.2	88.1	55.0
w/ $L_{\mathrm{FM}}$ only	$82.5_{+1.8}$	$86.5_{+1.0}$	$70.6_{+1.6}$	$69.7_{+1.5}$	$89.2_{+1.1}$	$55.7_{+0.7}$
$L_{\mathrm{FM}}+\mathrm{FreqT}$	$83.0_{+2.3}$	$86.6_{+1.1}$	$70.9_{+1.9}$	$72.5_{+4.3}$	$90.3_{+2.2}$	$56.2_{+1.2}$
$L_{\mathrm{FM}}+\mathrm{FreqS}$	$82.7_{+2.0}$	$86.8_{+1.3}$	$71.1_{+2.1}$	$71.8_{+3.5}$	$91.2_{+3.1}$	$55.9_{+0.9}$
$\mathrm{FreqS}+L_{\mathrm{FM}}+\mathrm{FreqT}$ (ours)	$84.2_{+3.5}$	$87.8_{+2.3}$	$71.7_{+2.7}$	$75.4_{+7.2}$	$91.8_{+3.7}$	$56.3_{+1.3}$

**Table 3:** Ablation study multiple analyses of model complexity on the proposed models. Acc denotes classification accuracy, #Params refers to the number of model parameters, and ∼FLOPs represents approximate floating-point operations. w/ and w/o mean with and without the corresponding module. + and ✓ indicate the inclusion of the corresponding component, while **✗** indicates its removal. $W_{g}$ and $W_{l}$ represent the global and local frequency filters, respectively.

Spatial–Temporal Aug. (NTU-60-Xsub-J)				Semantic Compensation (NTU-60-Xsub-J)			
Method	Acc (%)	#Params	^∼^FLOPs	Method	Acc (%)	#Params	^∼^FLOPs
SkeleMixCLR [29]	80.7	1.90 M	^∼^1.70 G	SkeleMixCLR [29]	80.7	1.70 M	^∼^1.72 G
+$L_{FM}$	$82.5_{+1.8}$	1.90 M	^∼^1.70 G	+$L_{M}$, *FreST*	$84.2_{+3.5}$	2.08 M	^∼^1.72 G
✓ frequency spatial	$82.7_{+2.0}$	1.90 M	^∼^1.70 G	**✗** $W_{l}$	$83.7_{-0.5}$	2.08 M	^∼^1.72 G
✓ frequency temporal	$83.0_{+2.3}$	1.90 M	^∼^1.70 G	**✗** $W_{g}$	$83.5_{-0.7}$	2.08 M	^∼^1.72 G
CrosSCLR [28]	77.8	2.45 M	~2.10 G	ActCLR [47]	80.9	2.01 M	~1.72 G

**Table 4:** Linear evaluation and finetune results on NTU-60 and NTU-120. Numbers in blue and red reflect improvement and decline compared to SkeletonCLR [28], AimCLR [46], and ActCLR [47] with the same backbone, respectively.

Stream	Method	NTU-60		NTU-120	
		X-Sub	X-View	X-Sub	X-Set
Linear Evaluation Protocol					
Single-stream	SkeletonCLR	68.3	76.4	56.8	55.9
	AimCLR	74.3	79.7	63.4	63.4
	ActCLR	80.9	86.7	69.0	70.5
	**MICA**	$84.2_{+3.3}$	$87.8_{+1.1}$	$71.7_{+2.7}$	$75.4_{+4.9}$
Multi-stream	3s-CrosSCLR	77.8	83.4	67.9	66.7
	3s-AimCLR	78.9	83.8	68.2	68.8
	3s-ActCLR	85.1	91.4	75.4	76.0
	**3s-MICA**	$85.3_{+0.2}$	$90.6_{-0.8}$	$77.4_{+2.0}$	$76.0_{+0.0}$
Finetune Evaluation Protocol					
Single-stream	SkeletonCLR	82.2	88.9	73.6	75.3
	AimCLR	83.0	89.2	76.4	76.7
	ActCLR	85.8	93.9	79.4	80.9
	**MICA**	$86.0_{+0.2}$	$92.5_{-1.4}$	$78.2_{-1.2}$	$80.6_{-0.3}$
Multi-stream	3s-CrosSCLR	86.2	92.5	80.5	80.4
	3s-AimCLR	86.9	92.8	80.1	80.9
	3s-ActCLR	88.2	93.9	82.1	84.6
	**3s-MICA**	$88.3_{+0.1}$	$94.1_{+0.2}$	$82.3_{+0.2}$	$84.9_{+0.3}$

**Table 5:** Semi-supervised evaluation results on NTU-60 and NTU-120 dataset. The best scores are shown in bold.

Method	NTU-60		PKU-MMD	
	X-Sub	X-View	Part I	Part II
1% labeled data for semi-supervised evaluation:				
$MS^{2}L$ [38]	35.2	-	36.4	13.0
3s-CrosSCLR [28]	51.1	50.0	49.7	10.2
3s-AimCLR [46]	54.8	54.3	57.5	15.1
3s-CMD [67]	55.6	55.5	-	-
**3s-MICA (Ours)**	$56.0_{+0.4}$	$55.8_{+0.2}$	$62.9_{+5.4}$	$17.5_{+2.4}$
10% labeled data for semi-supervised evaluation:				
$MS^{2}L$ [38]	65.2	-	70.3	26.1
3s-CrosSCLR [28]	74.4	77.8	82.9	28.6
3s-AimCLR [46]	78.2	81.6	86.1	33.4
3s-SDS-CL [72]	77.2	83.0	-	-
3s-CMD [67]	79.0	82.4	-	-
3s-SelMixCLR [29]	79.9	83.6	87.7	41.0
**3s-MICA (Ours)**	$80.2_{+1.2}$	$85.0_{+2.0}$	$88.6_{+2.5}$	$42.2_{+1.2}$

**Table 6:** A ablation study of MICA different CLIP text encoders streaming a NVIDIA 3090 on an ntu-60 xsub joint stream connector with batch size set to 64.

CLIP Text Encoder	Pre-Train	Acc@1 (%)	Memory (GB)
VIT/B-32	text/img	84.40	23.12
VIT/B-16	text/img	84.33	23.12
VIT/L-14	text/img	83.26	23.12
ResNet-50	text	83.67	23.12

## Data Availability

Restrictions apply to the availability of these data. Data were obtained from Rose Lab and are available https://rose1.ntu.edu.sg/dataset/actionRecognition/ (accessed on 27 April 2022) with the permission of Rose Lab.
